# A Health Records Review of Outpatient Referrals from the Emergency Department

**DOI:** 10.1155/2019/5179081

**Published:** 2019-10-31

**Authors:** Nicholas Prudhomme, Edmund S. H. Kwok, Laura Olejnik, Shannon White, Venkatesh Thiruganasambandamoorthy

**Affiliations:** ^1^Department of Emergency Medicine, University of Ottawa, Ottawa, ON, Canada; ^2^Ottawa Hospital Research Institute, The Ottawa Hospital, Ottawa, ON, Canada

## Abstract

**Objectives:**

Many patients discharged home from the emergency department (ED) require urgent outpatient consultation with a specialty service. We sought to identify the best- and worst-performing services with regard to time to outpatient consultation, the proportion of patients lost to follow-up, the rate of related return ED visits prior to consultation, and common strategies used by our top-performing clinics.

**Methods:**

We conducted a health records review of The Ottawa Hospital ED visits during four 1-week periods. All consecutive adult outpatient consultation requests were included for chart review and were followed up to 12 months. Outcome measures included demographics, referral attendance rates, incomplete referrals, return ED visits, and time intervals. Services with at least 15 consultation requests were included for data analysis and qualitative mapping of their referral processes.

**Results:**

Of the 963 patients who met inclusion criteria, 803 (83.4%) attended their appointment, while 160 (16.6%) were lost to follow-up. The overall median time to successful consultation was 9 days (IQR = 2–27). 92 (9.6%) patients returned to the ED with a related complaint. The top-performing clinics included ophthalmology, orthopedics, and thrombosis (median = 1, 8, 1 days; incomplete consultation = 3%, 4%, 6%; return ED visits = 0%, 6%, 2% respectively). The bottom-performing clinics included otorhinolaryngology, neurology, and gynecology (median = 47, 39, 27 days; incomplete consultation = 50%, 41%, 37%; return ED visits = 11%, 15%, 26%, respectively). Processes incorporated by top-performing clinics included reserving appointment slots for emergency referrals, structured referral forms, and centralized booking.

**Conclusions:**

We found a substantial variability in both the waiting times and reliability of outpatient referrals from the ED. Top-performing clinics incorporate common referral processes.

## 1. Introduction

The majority of patients presenting to the emergency department (ED) are discharged home without needing hospitalization; however, some require urgent outpatient specialist consultation. Outpatient consultations from both EDs and family physicians are increasing, rising from 4.8% to 9.3% from 1999 to 2009 in the United States [1]. Patients are harmed by delays to outpatient care, miscommunications among care providers, and loss to follow-up. Alberta's Continuity of Patient Care Study was inspired by the unfortunate death of a patient following a series of miscommunications and delays to care [2]. This report identified a series of patient-centred recommendations to improve reliability and prioritization of time-sensitive outpatient consultations, including the incorporation of referral tracking systems and expedited diagnostic testing sequences [2].

Currently, outpatient referrals from our ED are made via a complex series of manual steps which can be unreliable and negatively impact quality of care. Many highly variable processes are in place to both communicate referrals and book consultation appointments. Recently, a review of patients with unfavourable outcomes during quality improvement rounds at our centre highlighted the negative impact on patient safety due to the diverse and unreliable nature of outpatient referral processes. Additionally, there are no local data on wait times for outpatient consultation for ED patients and the proportion of patients who complete the consultation process. This impedes our ability to provide reliable follow-up expectations upon discharge from the ED. Previous studies have not reported both time to consultation and rate of loss to follow-up for ED patients referred for outpatient consultation. Further, there is limited literature identifying the effects of outpatient referral processes on these outcomes, specifically the presence of structured referral forms, centralized booking systems, and prespecified ED-specific appointment slots.

Our objectives were to quantify the time to outpatient consultation, the rate of return ED visits for related complaints prior to their intended appointment, and the proportion of patients lost to follow-up for a cohort of ED patients referred to a range of outpatient specialty services from a tertiary care ED. We sought to identify the best- and worst-performing services for qualitative analysis to highlight the outpatient referral processes common to the most reliable clinics.

## 2. Methods

### 2.1. Study Design and Setting

We conducted a health records review of patients presenting to The Ottawa Hospital Emergency Department. The Ottawa Hospital is a tertiary care academic hospital with approximately 160,000 ED visits per year. It is a teaching hospital affiliated with the University of Ottawa, and its two sites provide care primarily to adult patients. Paper charts are still used in the EDs; therefore, outpatient consultation requests are written on a consultation form. An ED clerk collects these forms, which are faxed or physically transported to the intended outpatient clinic. Consultation forms are then scanned into the electronic medical record (EMR), Open Architecture Clinical Information System (vOACIS™). Individual services can have unique booking processes: patients may be required to call and book their own appointments; services may incorporate a centralized triage and booking system, while others are faxed to the on-call consultant; some services reserve appointment slots specifically for the rapid assessment of ED patients; clinic-specific referral forms are occasionally in use. Outpatient consultations are then documented in vOACIS in the form of a written or dictated report. Further, a separate outpatient booking Shared Medical System (SMS) can be used to track undocumented consultation visits. This study was approved by the Ottawa Hospital Research Institute's research ethics board.

### 2.2. Population

We enrolled patients who presented during four distinct one-week periods: the first weeks of October 2014, January 2015, April 2015, and July 2015 were included. These periods were chosen to account for seasonal variations in ED outpatient consultations. All adult (age ≥18) patients for whom an outpatient consultation was requested by an ED physician during the study periods were included for chart review. These patients were identified via a manual review of all consultation request forms scanned into vOACIS™. Patients were excluded if they were referred to a specialty clinic external to our academic centre, as their consultation would not have been logged in vOACIS. Out-of-province patients were excluded if they were inadvertently referred to a clinic with an active policy in place to refuse and redirect such patients.

The charts of all patients who met the above criteria were reviewed for up to 12 months postindex ED visit. Chart review and data abstraction were performed by two study investigators (NP and LO). We abstracted patient demographics, the service consulted, and the reason for referral.

### 2.3. Outcome Measures

Primary outcomes included the proportion of patients lost to follow-up, the days to the initial outpatient consultation, and the number of return ED visits with a related chief complaint prior to initial outpatient appointment. Time to consultation was determined by abstracting the date the consultation occurred as logged in vOACIS™ or by using the consultant's report. In the event no such report was found, we cross-referenced the patient data with the SMS booking system to ensure that no appointment was logged without documentation on vOACIS™. Patients were deemed lost to follow-up if neither documentation of consultation nor a logged appointment was present.

10% of all charts were reviewed independently by two investigators (NP and LO) for assessment of the inter-rater reliability of data abstraction and outcomes assessment. To enhance the validity of our findings, we decided *a priori* to exclude the patient data referred to outpatient specialty services with infrequent consultations, less than 15 over the study period.

### 2.4. Data Analysis

We used median and interquartile range to describe continuous data that are skewed and proportions for categorical data with 95% confidence intervals when appropriate. We report inter-rater reliability using kappa statistics and percentage proportional agreement.

The top- and bottom-performing services for all primary outcomes were identified. All clinics with at least 15 consultations in the study period underwent process mapping. ED clerks and booking clerks for each clinic were contacted to include all major components of the outpatient consultation scheduling process, including the presence of dedicated referral forms, rapid-access appointment slots for urgent ED referrals, patient communication requirements, and whether consults were triaged after being sent to a centralized triage system versus the specific on-call consultant.

## 3. Results

**Figure 1:**
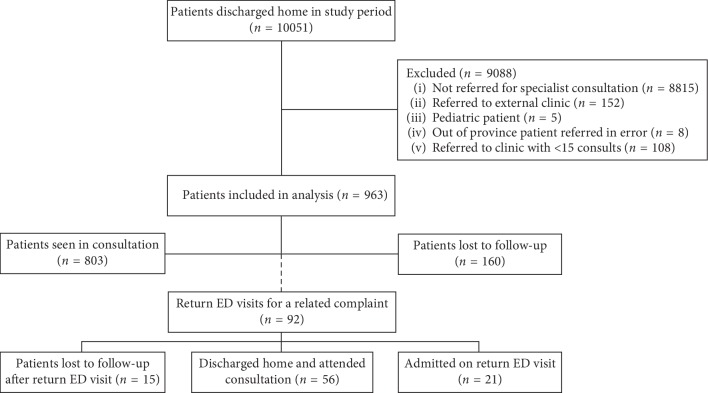
Flow chart of patients discharged from the ED with outpatient consultations.

A total of 10,051 patients were seen and discharged from the ED during the study periods. Outpatient specialist consultations were requested for 1,236 (12.3%) patients.  Figure 1 depicts a flowchart of these patients' outpatient referral process. Of these consultations, 165 (13.3%) met exclusion criteria as identified in Figure 1. 18 outpatient clinics with 108 consultations (8.7% of the total) were excluded from analysis, as they each had fewer than 15 consultations during the study period. This left 963 patients for analysis. Baseline demographic information is presented in Table 1.

**Figure 2:**
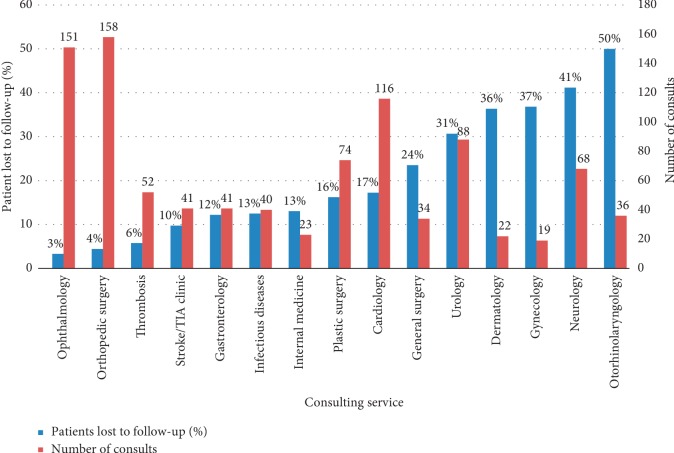
Outpatient consultations and patients lost to follow-up for frequently consulted services for emergency department patients.

Overall, 160 (16.6%) patients included in the analysis were lost to outpatient follow-up (Table 1). The median time to consultation was 9 days (IQR 2–27). 92 (9.6%) patients returned to the ED for a related complaint prior to their intended consultation. The ophthalmology and orthopedic surgery services received the greatest number of referrals (Figure 2).

Ophthalmology, orthopedic surgery, and thrombosis had the fewest patients lost to follow-up (Figure 2). Conversely, gynecology, neurology, and otorhinolaryngology had the greatest losses to follow-up. 110 patients were reviewed independently by both investigators (NP and LO). The calculated kappa coefficient was 1 (95% CI N/A) for inter-rater agreement for lost to follow-up.

**Figure 3:**
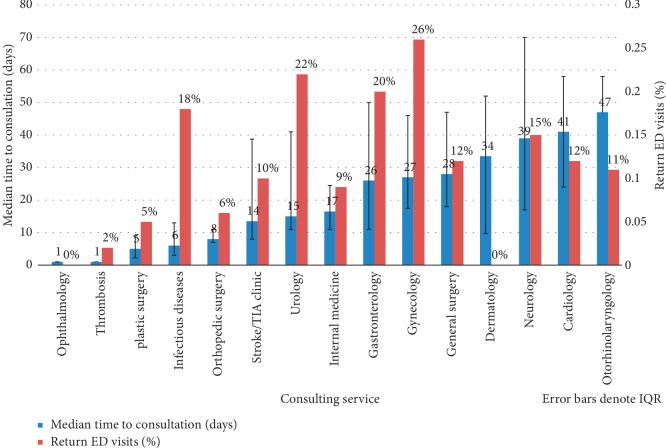
Rate of return ED visits and time to consultation for frequently consulted services for emergency department patients.

Regarding the time to outpatient consultation, Figure 3 demonstrates that ophthalmology, thrombosis, and plastic surgery were the fastest to see patients. Neurology, cardiology, and otorhinolaryngology had longer, more variable wait times. The proportional agreement between both reviewers was calculated to be 93.6%.

92 (9.6%) patients returned to the ED with a related complaint; of these, 21 (2.2%) required admission (Figure 1). Figure 3 demonstrates that the ophthalmology, dermatology, and thrombosis clinics had the fewest return ED visits. Conversely, gastroenterology, gynecology, and urology had the greatest rate of return ED visits. Of these, 3 gastroenterology, 1 gynecology, and 4 urology patients required admission.

Table 2 depicts the key elements of each specialty clinic's referral process. Top-performing clinics frequently incorporated rapid appointment slots dedicated to ED patients. Clinics without centralized intake systems generally performed less reliably. The use of dedicated referral forms was infrequent, although those who incorporated them often performed more reliably and expediently. Requiring patients to contact the clinics to book their own appointments did not seem to be associated with the reliability of consultation.

## 4. Discussion

Of our ED patients discharged home with an outpatient specialist referral, 16.6% were lost to follow-up and 9.6% returned to the ED with a related complaint. This moderate proportion of patients lost to follow-up is important to explore, given the significant fraction of patients discharged home with the intention of expedient specialist consultation. The overall median time to consultation was 9 days, although this was highly variable (IQR 2–17). Importantly, we observed a significant degree of variability among our specialty clinics for all of our outcomes of interest. Similarly, referral booking processes were often unique to each clinic which may have impacted the reliability and timeliness of follow-up for each clinic. The potential impact of these referral processes will be explored below.

Referrals to specialists from ambulatory care settings continue to increase [1]. Our cumulative referral rate of 12.3% is similar to that of one Australian ED-specific follow-up study, which noted an outpatient referral rate of 16.3% [3]. There is a large variation in reported proportions of patients lost to follow-up in the emergency medicine literature, ranging from 24 to 63% [3–5]. Our global finding that 16.6% of referred patients were lost to follow-up is reasonable by comparison. However, 32.1% of our patients were referred to our two most reliable specialist clinics, thereby potentially biasing our cumulative results in favour of reliable consultation.

We observed an expedient yet variable median time to specialist consultation of 9 days. Recent Canadian data have demonstrated a median wait time from family physician referral to specialist appointment of 71 days among all provinces and 47 days for Ontario alone [6]. Our results highlight that our best-performing services are both expedient and reliable with the fewest return ED visits; with long delays to consultation, it is possible that patients decide to seek care elsewhere, or their presenting complaint has diminished to the point that it is no longer a priority.

Our ophthalmology, thrombosis, orthopedic surgery, and plastic surgery clinics all have dedicated rapid appointment slots for ED referrals. As part of a bundle of measures to improve both inpatient and outpatient specialist access blocks, one institution mandated same or next day emergency appointment slots for all specialist outpatient clinics, with prespecified referral guidelines for optimal triage [7]. Subsequently, the proportion of patients seen within their requested time frame rose from 51.7% to 80.8%. Further, a recent systematic review of ED referral processes demonstrated that the provision of a consultation appointment prior to ED departure was one of the greatest predictors of increasing consultation completion [8].

The highest proportion of incomplete referrals in our study was found among our neurology and otorhinolaryngology services. Neither fully incorporates centralized referral processing, which may present an increased risk of misplacing consultation requests in transit from the ED to the outpatient clinic. Centralized intake systems have shown benefits in the literature; one study demonstrated reduced wait times for moderate and urgent referrals to rheumatologists while improving referral quality and eliminating duplicate referrals [9].

Our thrombosis, stroke, cardiology, and infectious diseases clinics all utilize unique referral forms which require specific criteria to triage and plan subsequent testing. The literature focusing on family physician referrals to specialists have highlighted that structured referral forms are effective at improving referral appropriateness [10]. Although infrequently incorporated by the clinics in this study, the observed correlation between structured referral forms and reliability of consultation complements the existing literature.

We found a lost to follow-up rate of 17% for referrals to cardiology; by comparison, that of a recent Canadian study was 10.4% [11]. This observed difference may be explained by the latter study's robust call-back process, which is garnering support in the literature. One randomized control trial found that a single telephone call 1–3 days post-ED visit improved consultation attendance rates from 54.4% to 70.7% [3]. Another demonstrated an improvement in follow-up with any medical provider, from 37% to 54%, following a similar intervention [12]. The impact of post-ED visit call-backs on return ED visits is less consistent; a recent systematic review highlighted conflicting results when assessing the outcome of such call-backs on either 30- or 60-day return of ED visits [8]. More recently, no improvement was found for the same intervention on 30-day return visits or hospitalizations for patients aged 65 years and over [13]. While not examining post-ED visit call-backs explicitly, our study did not observe an effect on any of our outcome measures when the clinic itself calls the patient to book their consultation. In the population of patients referred for outpatient consultation, it remains unclear to what degree post-ED visit telephone calls affect return ED visits.

### 4.1. Recommendations

Our study complements the literature in demonstrating the ED's high reliance on outpatient consultation; it is critical that outpatient consultation for ED patients is obtained in a timely and reliable manner. We have identified the top-performing clinics in these domains and have explored the individual processes employed by each clinic to better inform the optimization of outpatient specialist referrals, which remains sparse in the literature. The ideal solution to streamline specialist care may lie in reserving rapid-access specialist appointment slots for select, high-risk patients from the ED. Centralized consultation processing systems also appear to feature prominently in delivering timely and reliable care to discharged ED patients. Whether by limiting the referral paper trail or by eliminating duplicate and inappropriate referrals, central processing systems are becoming the standard of care. Further, the literature also supports a role for ED call-back processes in optimizing consultation completion provided they are economically and logistically feasible.

While less well studied in the literature, incorporating dedicated referral forms highlighting pertinent clinical data and urgency may represent an opportunity to optimize specialist care for discharged ED patients. It is possible that such forms screen for appropriate ED referrals, resulting in earlier appointments and improved consultation completion rates via objective prioritization and decreased rates of inappropriate referrals.

Following the completion of this study, our centre has since incorporated a new electronic medical record which is capable to centralized specialist referral processing. Many clinics now require specific information to improve consultation triage.

### 4.2. Limitations

The single-centred nature of our study limits the generalizability of our findings; the variation in each specialty's timeliness and reliability may be the result of local resource limitations. At the time of this study, our centre lacked an electronic medical record with a centralized referral tracking system; therefore, to accurately and feasibly capture all consultations from the ED, we limited our review to consecutive referrals during specific time periods. Infrequently consulted services are subsequently under-represented with few consults and were not included for analysis. Further, our sample may not account for subtle seasonal variations on consultation requests and delays. While delays to consultation can impact the patient experience, we acknowledge that patients often cancel or do not present to their appointment of their own accord. Our methodology was not able to reliably capture these occurrences as they were inconsistently recorded in our EMR.

## 5. Conclusion

Our current state analysis demonstrates a substantial variability in outpatient consultation wait times and reliability. Specialist clinics with the fewest incomplete referrals also booked patients most expediently, with infrequent return ED visits. Processes incorporated by top-performing clinics included reserving appointment slots for emergency referrals, structured referral forms, and centralized consultation booking. Hospital systems should develop standardized best-practice guidelines to optimize access to outpatient specialists.

## Figures and Tables

**Table 1:** Patient characteristics and summative results (*N* = 963).

Demographics	
Mean patient age (years)	51
Female sex (%)	49.5

Outpatient clinic	Patients referred (*n*)
Cardiology	116
Dermatology	22
Gastroenterology	41
General surgery	34
Gynecology	19
Infectious diseases	40
Internal medicine	23
Neurology	68
Ophthalmology	151
Orthopedic surgery	158
Otorhinolaryngology	36
Plastic surgery	74
Stroke/TIA clinic	41
Thrombosis	52
Urology	88

Summative results	
Patients lost to follow-up	160 (16.6%)
Overall median days to consultation	9 (IQR 2–27)
Return ED visits for a related complaint	92 (9.6%)

IQR, interquartile range.

**Table 2:** Qualitative analysis of outpatient referral processes from the emergency department.

Consulting service	Patient to contact clinic	Dedicated referral form	Centralized intake	Rapid ED appointments
Ophthalmology	✗	✗	✓	✓
Orthopedic surgery	✓	✗	✓	✓
Thrombosis	✗	✓	✓	✓
Stroke/TIA clinic	✗	✓	✓	✗
Gastroenterology	✗	✗	✓	✗
Infectious diseases	✗	✓/✗	✓/✗	✗
Internal medicine	✗	✗	✓	✗
Plastic surgery	✓	✗	✓	✓
Cardiology	✓/✗	✓	✓	✗
General surgery	✗	✗	✓	✗
Urology	✗	✗	✗	✗
Dermatology	✗	✗	✓	✗
Gynecology	✗	✗	✓	✗
Neurology	✗	✗	✓/✗	✗
Otorhinolaryngology	✓	✗	✗	✗

Clinics are presented from top to bottom in ascending order of proportion of patients lost to follow-up. Combinations of tick marks and x's indicate that there are two internal outpatient clinics, one for each campus, which have different processes for booking consultations.

## Data Availability

Deidentified data are available upon request.
